# Modified Crushed Oyster Shells for Fluoride Removal from Water

**DOI:** 10.1038/s41598-020-60743-7

**Published:** 2020-04-01

**Authors:** Woohang Kim, Rekha Singh, James A. Smith

**Affiliations:** 1 Department of Environmental Engineering & Biotechnology, Mokpo National Maritime University, 91 Haeyangdaehak-ro, Mokpo, 58628 South Korea; 2 Department of Engineering Systems and Environment, University of Virginia, Charlottesville, VA 22904 USA; 3 Department of Engineering Systems and Environment, University of Virginia, Charlottesville, VA 22904 USA

**Keywords:** Environmental sciences, Natural hazards, Materials science

## Abstract

Elevated concentrations of fluoride ions (F^−^) in natural groundwater are a worldwide problem. Discarded oyster shells were ground to ≤100 µm particle size to produce oyster shell powder (OS). A subset of the OS was heated to produce calcined oyster shell (COS). A subset of the COS was further treated with 1 M phosphoric acid to produce phosphoric-acid-treated oyster shell (POS). OS and COS were combined with phosphoric acid (1.6 mM and 3.2 mM) to produce OS + P (oyster shell with phosphoric acid) and COS + P (calcined oyster shell with phosphoric acid). OS and COS removed 46% and 50% (10 g/L of sorbent dose) but POS, OS + P and COS + P removed 96%, 100% and 76% (1 g/L of sorbent dose) when the initial concentration of fluoride was 10 mg/L. The sorption kinetics of POS, OS + P and COS + P followed second-order reaction rates, and sorption isotherms of all sorbents were well-described by the Freundlich sorption isotherm. These results indicate that oyster shells can be an effective sorbent for fluoride removal, with the added benefit of re-use of a waste product.

## Introduction

Fluoride (F^−^) concentration in drinking water is an important parameter to determine water quality for drinking purposes^1^. Fluoride in drinking water has both beneficial and detrimental effects, depending on its dose. At low concentrations, it protects against tooth decay^2^. By contrast, the ingestion of water with a fluoride concentration above 1.5 mg/L causes dental and skeletal disorders^3^. Groundwater in many areas around the world has high levels of fluoride concentration^4^. The World Health Organization (WHO)^4^ recommends a fluoride concentration of less than 1.5 mg/L in drinking water. Because some groundwater naturally has high levels of F^−^, millions of people worldwide are affected by drinking this water. A study by UNICEF reported that fluorosis is endemic in more than 25 countries around the world. The natural concentration of fluoride in groundwater depends on geological characteristics of the aquifer, the physical and chemical characteristics of soil and rock, and depth of the wells. All these factors contribute to variable concentration of F^−^, which generally ranges between 1 mg/L and 44 mg/L, throughout the world^5^.

Many technologies, based on sorption, ion exchange, chemical precipitation, reverse osmosis and electro dialysis have been used to remove excessive fluoride from drinking water. Among them, sorption is one of the most common technologies because of ease of operation and cost effectiveness^4^. Many materials such as hydroxyapatite, activated alumina, limestone, activated carbon, zeolite, calcite, and clay have been used for fluoride sorption^6^. Low cost materials such as limestone, quick lime, bentonite, kaolinite, china clay and calcite have been tested for fluoride removal and proven to be effective^7^. However, at low F^−^ concentrations, these materials lose their fluoride removal capacity and they are not suitable for drinking water because of limited fluoride sorption^8^. So it is important to identify new materials that are inexpensive and efficient for fluoride removal. We hypothesize that oyster shells meet these design criteria for fluoride removal from water.

Oyster shells are produced in large amounts from the marine food industry in China as well as in some other countries^9^. They can potentially be used as an inexpensive material for water treatment. Oyster shells are a rich source of calcium carbonate^10–12^ that can be used for fluoride removal^13^. Calcium carbonate can be used to produce hydroxyapatite which has been shown to be effective for fluoride removal from water^14^. Phosphoric acid–enhanced limestone has improved fluoride removal efficiency and is a low-cost material^15^. In composition, oyster shells resemble limestone and therefore have the potential for significant fluoride removal from water. However, to date, no studies have investigated the equilibrium and kinetic sorption of fluoride to oyster shells treated to optimize performance.

This work studies the feasibility of using oyster shells as a sorbent for fluoride removal from water. The sorption of fluoride by different forms of oyster shell fragments from aqueous solution was measured in a series of equilibrium and kinetic batch experiments and for different water chemistries. To our knowledge, this is the first study to evaluate the use of waste oyster shells for F^−^ removal from water with consideration of thermodynamic and rate effects.

## Results and Discussion

### Sorption studies

**Figure 1:**
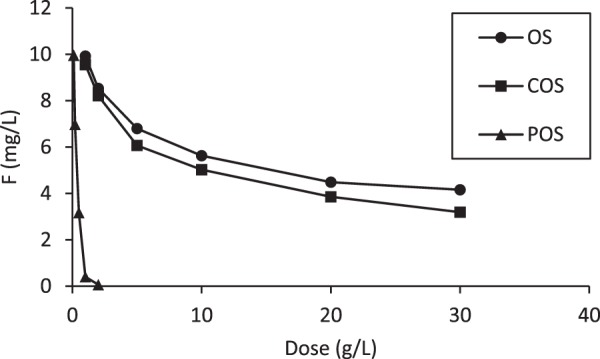
Concentration of fluoride with increasing sorbent dose after a 24-hr contact time (Sample volume 50 mL).

Figure 1 shows the aqueous F^−^ concentration as a function of increasing mass of sorbents for OS, COS and POS. The initial fluoride concentration was 10 mg/L. Fluoride concentration decreased with increasing dosages of each sorbent. 55% of fluoride was removed at 20 g/L of OS. The concentration of fluoride in batch reactors with COS was found to be slightly lower compared to reactors with OS. The concentration of fluoride in POS decreased very sharply and was less than 0.5 mg/L at 1 g/L of sorbent dose. It is evident that POS was an excellent sorbent compared to others.

Hydroxyapatite derived from limestone or oyster shells has the potential for significant fluoride removal from water. Hydroxyapatite that was produced by reaction between oyster shells and (NH_4_)_2_HPO_4_ at 30 °C completely removed 20 mg/L of fluoride^13^. But hydroxyapatites produced at 4, 60, and 120 °C did not completely remove fluoride.

**Figure 2:**
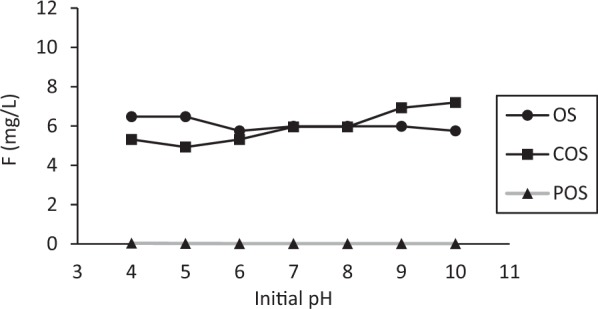
Concentration of fluoride as a function of pH (Sorbent dose 10 g/L of OS, COS and 2 g/L of POS, 10 mg/L of initial fluoride, contact time 24 hr, sample volume 50 mL).

The effect of pH on fluoride sorption are presented in Fig. 2. Fluoride removal efficiency for OS was not affected by variation in pH whereas fluoride removal efficiency for COS decreased with increasing pH. In COS, calcium oxide is formed through the calcination of OS so that calcium ion in water can remove fluorine by chemical reaction^16^. It is considered that fluoride removal was decreased due to competition by higher amount of hydroxyl ions with pH increasing. POS performed the best at all pH ranges. This result shows POS sorption was not pH-dependent in this experiment (pH range 4 to 10). Mondal and George^17^ reported fluoride adsorption was not affected by pH range 3 to 10.

Sorption data were fitted to the Freundlich sorption model:

$${q}_{e}={K}_{f}{{C}_{e}}^{1/n}$$

where

*q*_*e*_ is the sorbed fluoride concentration (mg/kg)

*K*_*f*_ is the Freundlich sorption constant (L/kg)

*C*_*e*_ is the equilibrium aqueous fluoride concentration (mg/L)

1/*n* is the sorption intensity constant (dimensionless)

**Figure 3:**
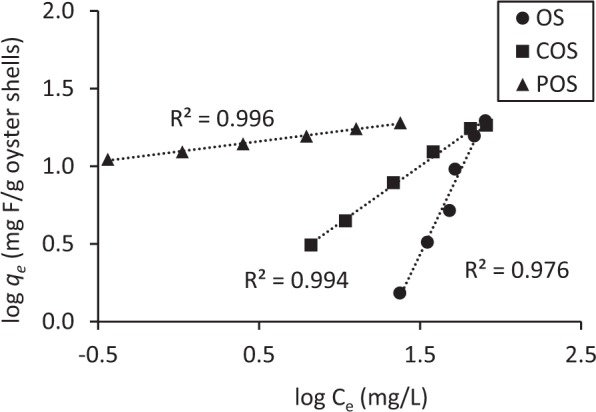
Freundlich sorption isotherm (Fluoride initial concentration is 100 mg/L, sorbent does ranges from 0.5 g/L to 50 g/L, contact time is 48 hr).

Freundlich sorption isotherms for OS, COS and POS are plotted in Fig. 3 for a 48-hr equilibration period. The initial concentration of fluoride was 100 mg/L and the sorbent dose ranged from 1 g/L to 10 g/L. Correlation coefficients were 0.97, 0.99 and 0.99 for OS, COS and POS, respectively. Freundlich sorption capacity constants, *K*_*f*_ (L/kg), were 0.0015 for OS, 0.79 for COS and 12.44 for POS. The sorption capacity constant of COS was higher than OS and sorption capacity of POS was much higher than OS and COS. The sorption intensity constants (1/*n*) were 2.16 for OS, 0.73 for COS and 0.13 for POS. Generally, a larger *K*_*f*_ indicates stronger adsorption. These results indicate that POS demonstrates the strongest fluoride sorption.

**Figure 4:**
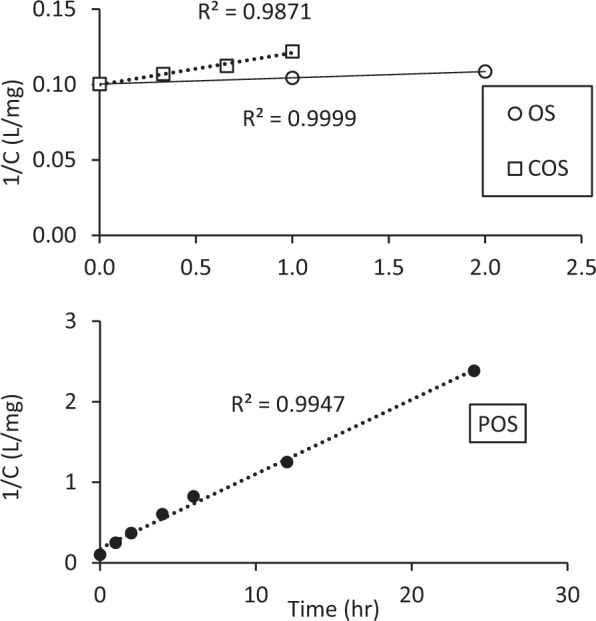
Second-order plot of fluoride sorption kinetics for OS, COS and POS at pH 7 and an initial concentration of 10 mg/L.

The rate of fluoride sorption was also investigated. The rate of sorption was determined by batch sorption kinetic experiments at an initial F^−^ concentration of 10 mg/L and a sorbent dose of 1 g/L (Fig. 4). OS and COS rate data were not plotted in the same graph because sorption equilibrium of OS and COS reached within 2 hours. Fluoride removal with all sorbents was described by the following second-order rate equation:

$$1/C=1/{C}_{o}+kt$$

where *k* (L/mg-hr) is the second-order reaction rate constant, *C* is the fluoride concentration at time *t*, and *C*_o_ is initial fluoride concentration. The sorption rate constants (*k)* were calculated from the slope of these graphs with 0.004, 0.021 and 0.092 at OS, COS, and POS.

### Characterization Study

**Figure 5:**
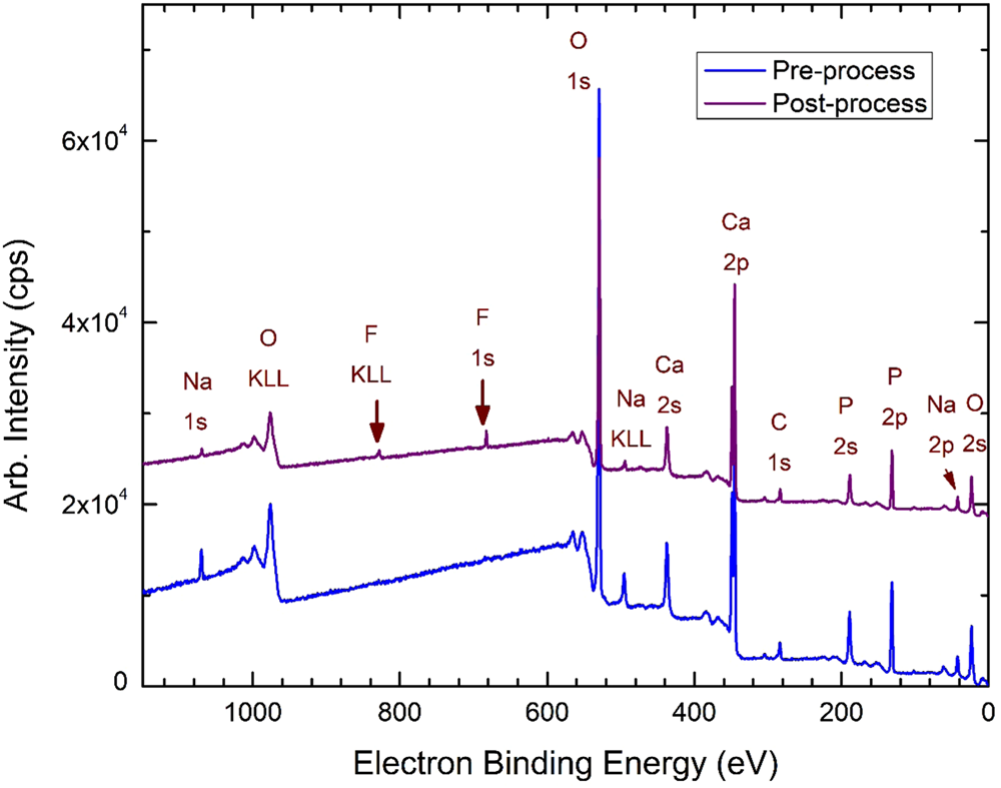
XPS spectra for pre and post sorption.

**Figure 6:**
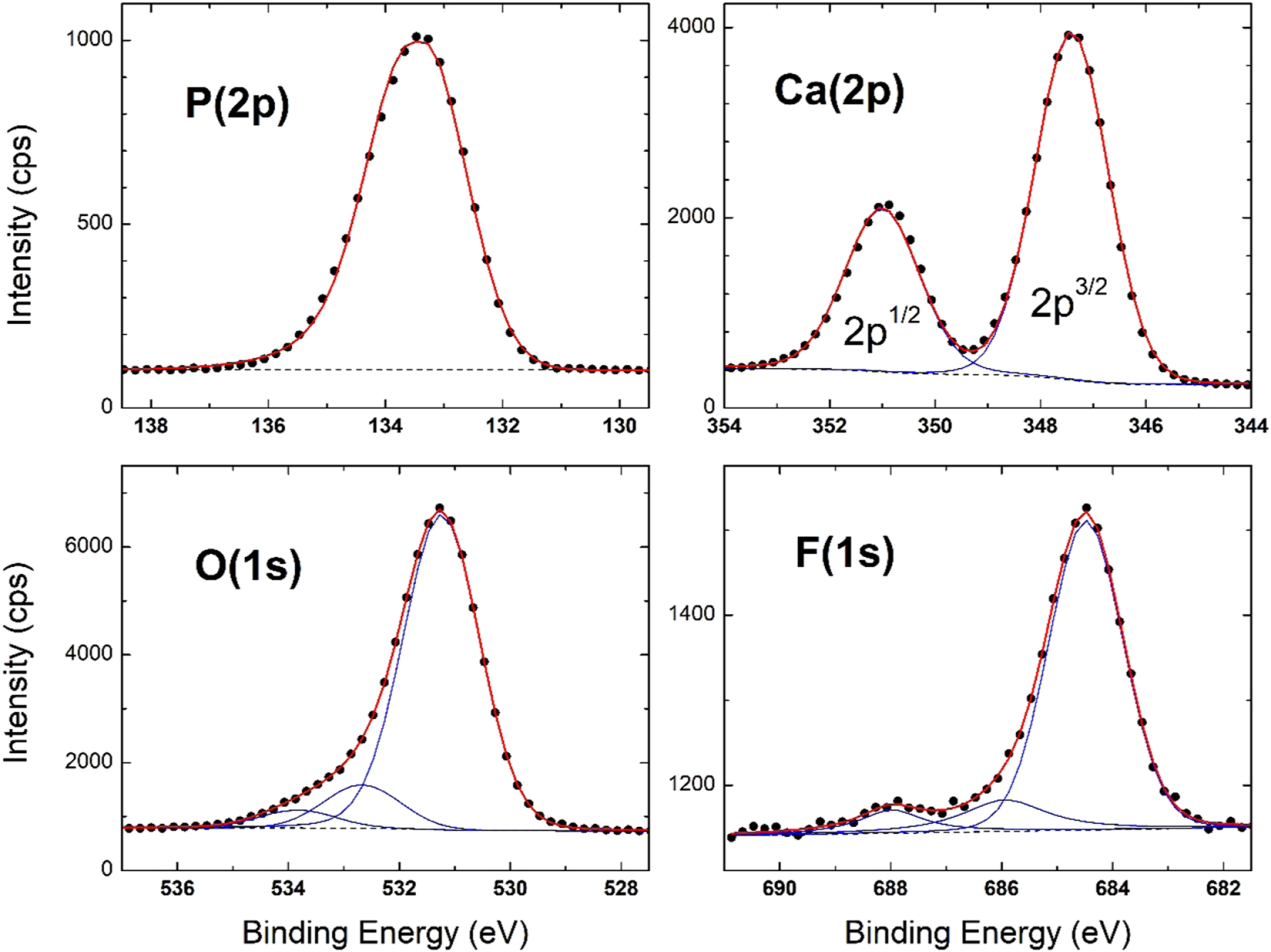
Quantification of survey spectra data; High resolution XPS spectra with binding energy of photo electrons.

**Figure 7:**
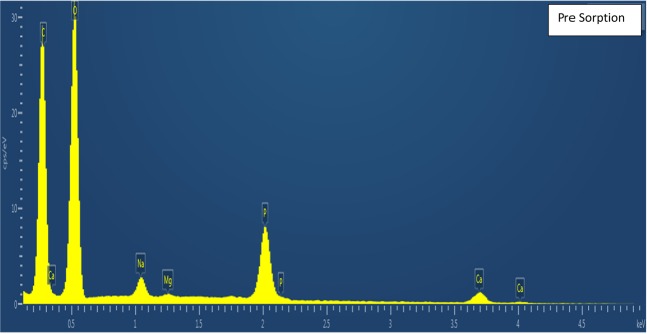
FESEM EDS spectra –Pre Sorption.

**Figure 8:**
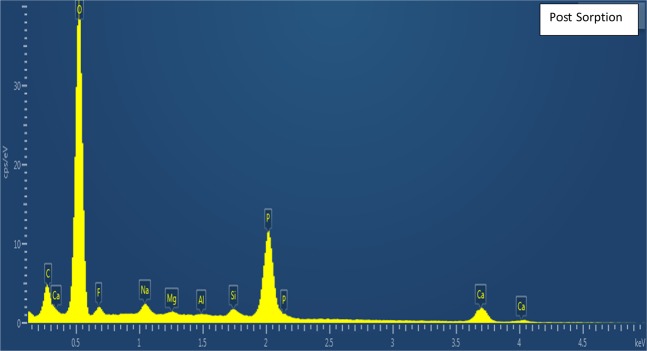
FESEM EDS spectra –Post Sorption.

**Figure 9:**
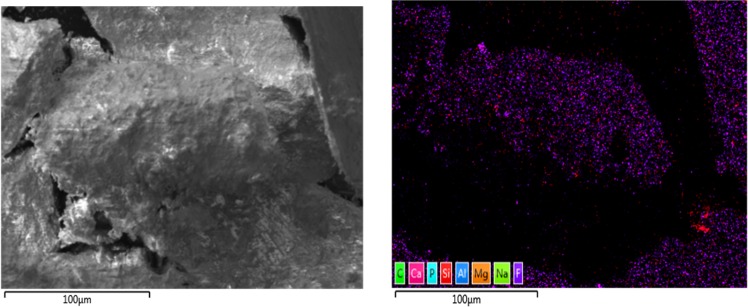
Heterogeneous distribution of F on the surface.

Material characterization was performed to confirm fluoride sorption and fluorapatite formation using XPS, SEMEDS and XRD analysis. There is no evidence of F in pre-sorption material, appearing only after-sorption in survey spectra **(**Fig. 5). This result is also confirmed by energy dispersive X-ray spectroscopy (EDS). In addition, high-resolution XPS spectra (Fig. 6) show that the adsorbed material surface chemistry is consistent with published values for fluroapatite^18^, based on binding energies for the F 1 s (684.6 eV), Ca 2p3/2 (347.5 eV), O1s (531.3 eV), and P2p (133.5 eV). FESEM-EDS spectra also shows presence of fluoride in post sorption samples, further confirming fluoride sorption (Figs. 7, 8). Also heterogeneous distribution of F was observed on the surface of the material (Fig. 9).

**Figure 10:**
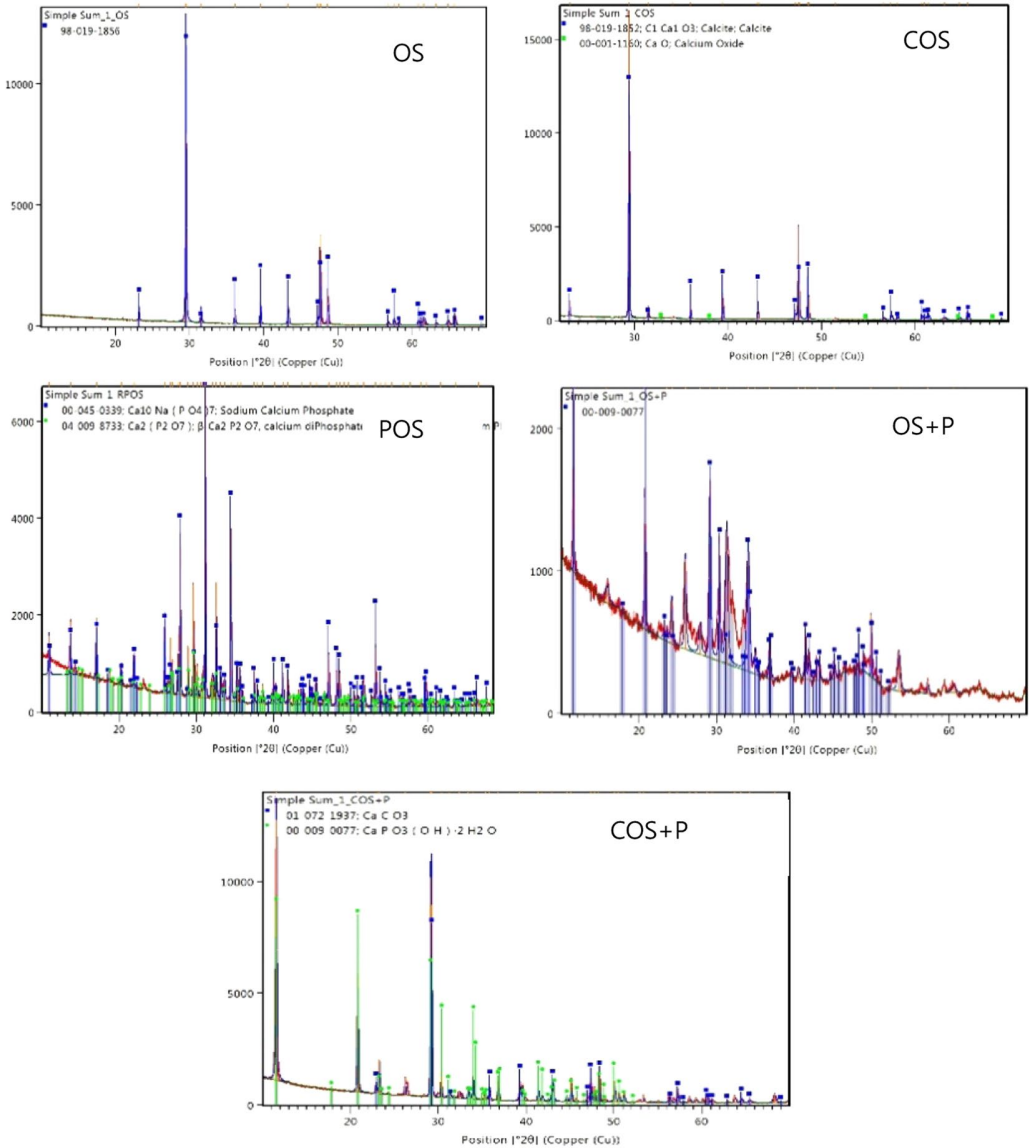
XRD pattern - chemical composition of various sorbents.

The XRD profiles of OS, COS, POS, OS + P and COS + P samples were examined by X-ray diffractometry (Fig. 10). The XRD pattern of OS and COS indicated that calcite (CaCO_3_) was the major crystal phase. However, phosphoric acid treatment of crushed oyster shell provoked deep changes in the chemical and crystal characteristics of the raw material, resulting in a POS mainly constituted by calcium phosphate hydroxide hydrate [CaPO_3_(OH)∙2H_2_O] and a lower amount of CaCO_3_. Also calcium phosphate was formed in OS + P treatment that is oyster shell reacted with phosphate in 10 mM of phosphoric acid.

**Figure 11:**
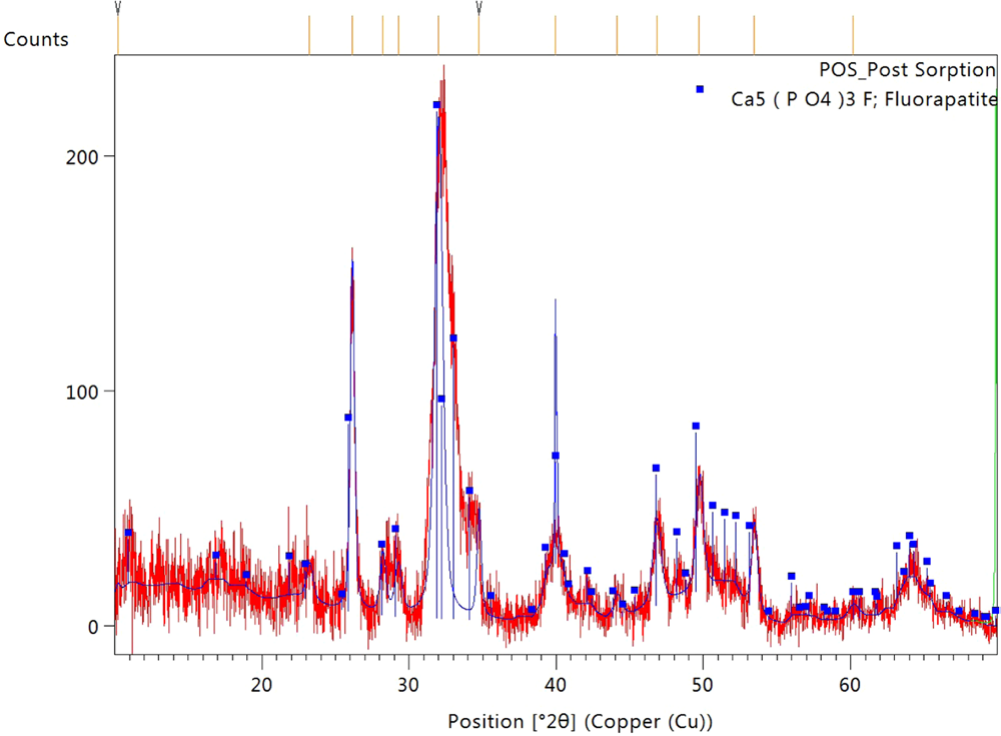
XRD pattern of post sorption as a confirmation of presence of Fluorapatite.

POS samples were analyzed to check whether hydroxyapatite is changing to fluorapatite after fluoride sorption. This is an evidence of fluoride removal as adsorbed material chemical composition is consistent with published values of fluorapatite (Fig. 11). Our sorbent is stable over a range of temperatures (0–700 °C).

Sludge was characterized for various physico-chemical parameters, cations and anions. pH was neutral for POS and slightly alkaline(pH 8) for OS. Only traces of F (0.5 mg/L) were detected. Relatively high concentration of phosphate (117 mg/L) was present whereas sulfate (1.33 mg/L) were very low if we compare with EPA drinking water limit (250 mg/L). The high concentration of phosphate is due to treatment with phosphoric acid but not from the material. It is recommended to rinse off the sorbent as a pre-treatment before using it. Also, the underlying hypothesis for this study was that the addition of phosphate will result in fluorapatite formation. To form fluorapatite, theoretically, 1 mole of fluoride is needed for 3 moles of phosphate. However, at a PA/F mole ratio of 3, 14% of the initial mass of phosphate remained in solution in OS + P. Other researchers have explained that the residual calcium (<1.50 mM) and phosphate (<8.40 μM) ions were found within the acceptable limit^15^.

### Addition of phosphoric acid

**Figure 12:**
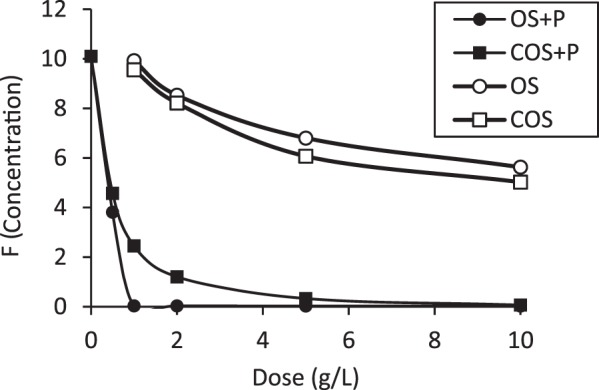
Equilibrium concentration of fluoride in phosphoric acid solutions in batch reactors as a function of different doses of OS and COS at 24 hr with (OS + P and COS + P) and without phosphoric acid addition.

**Figure 13:**
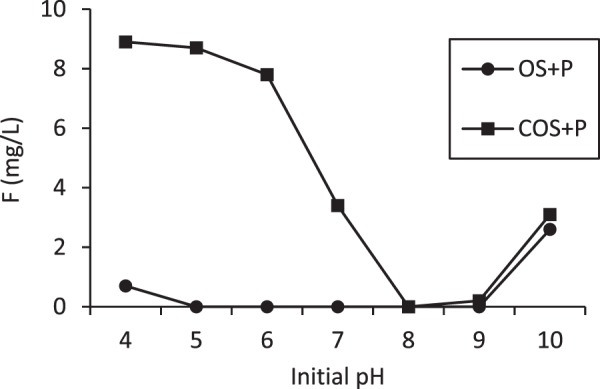
Concentration of fluoride with varying pH at constant dose of sorbents and phosphoric acid addition to the batch reactors.

**Figure 14:**
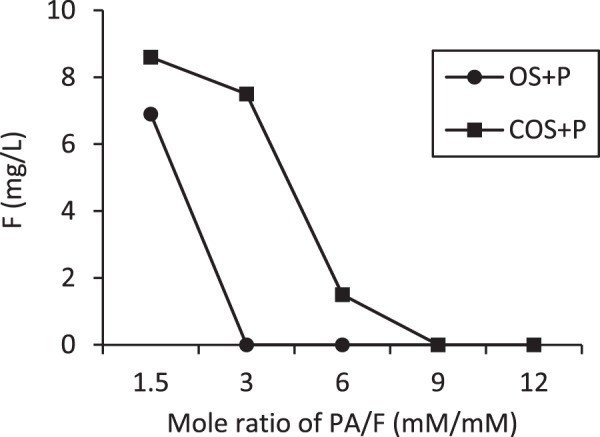
Concentration of fluoride as a function of the molar ratio of phosphoric to fluoride (PA/F) after a 24-hr equilibration period.

In order to increase fluoride removal, phosphoric acid was added to batch reactors containing OS and COS to facilitate formation of fluorapatite (Ca_5_(PO_4_)_3_F, or calcium fluorophosphate). Figure 12 compares fluoride sorption to OS and COS sorbents both with and without phosphoric acid addition. Phosphoric acid addition significantly increases fluoride sorption. As an example, the equilibrium fluoride concentration is about 8.2 mg/L with a 2 g/L COS dose, but the equilibrium concentration is less than 1.5 mg/L when phosphoric acid was added, which is well within the WHO recommended concentration for drinking water (1.5 mg/L). OS + P and COS + P removed 100% and 76% (1 g/L of sorbent dose) when the initial concentration of fluoride was 10 mg/L. Calcite can remove fluoride by both sorption and precipitation processes. The addition of phosphoric acid to the system results in formation of calcium phosphate and subsequent fluoride reaction to form fluorapatite^15^. The effect of pH on fluoride removal by OS + P and COS + P is shown in Fig. 13. Fluoride removal by OS + P was significant over a relatively wide pH range. It was completely removed in the pH range 5–9 at 10 mg/L of fluoride, 10 mM of phosphoric acid and a 24-hr treatment time. For COS + P, fluoride was almost completely removed at pH 8–9. The mechanism as we explained earlier for OS + P where there is hydroxyapatite (HA) formation is involved, there are two mechanisms involved in fluoride removal. First mechanism involved calcium (Ca^2+^) will be release and react with phosphoric acid to form hydroxyapatite and after exchanging F^−^ it get converted to fluorapatite. The other mechanism involved could be calcium from the sorbent react with phosphate and fluoride to form fluorapatite. Fluoride removal by COS + P was more sensitive to pH variation than OS + P. For COS + P fluoride removal was slow at low pH due to high solubility of calcium and there is no sorbent available to sorb fluoride. Fluoride removal was quantified as a function of phosphoric acid concentration (Fig. 14). An initial fluoride concentration of 10 mg/L (0.53 mM) was used and the phosphoric acid (PA) dose was adjusted to a PA/F mole ratio ranging from 1.5 to 12. For a PA/F mole ratio of 3, fluoride was not detectable in the aqueous phase for OS + P. A mole ratio of 9 was required to result in a non-detectable fluoride concentration for COS + P. In order to form fluorapatite, theoretically 1 mole of fluoride is needed for 3 moles of phosphate. However, at a PA/F mole ratio of 3, 14% of the initial mass of phosphate remained in solution for OS + P. Gogoi *et al*.^15^ reported that major fluoride removal mechanisms using phosphoric-acid-enhanced limestone were sorption of fluoride by the calcium phosphate and precipitation of CaF_2_.

**Figure 15:**
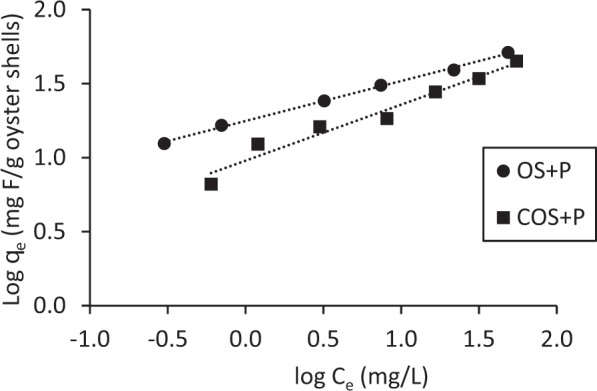
Freundlich sorption isotherms for two different sorbents with phosphoric acid addition (Fluoride concentration = 100 mg/L, OS and COS dose = 1 g/L to 15 g/L, temperature 20°C, contact time 48 hr).

Sorption data for OS + P and COS + P were effectively described by a Freundlich isotherm equation (Fig. 15). Correlation coefficients were 0.99 and 0.96 for OS + P and COS + P, respectively. The Freundlich sorption constant (*K*_*f*_) was 17.7 L/g for OS + P and 9.6 L/g for COS + P. Sorption intensity values (1/*n*) were 0.27 for OS + P and 0.38 for COS + P. Low sorption intensities correspond to relatively strong isotherm nonlinearity.

Freundlich constants for sorption isotherms are summarized in Table 1. The correlation coefficients range from 0.962–0.997. Sorption generally increases with increasing values of *K*_*f*_ and isotherm nonlinearity increases as deviations of 1/*n* from unity increase. Sorption intensity values greater than unity result in concave-up isotherms, whereas intensity values less than unity results in concave down isotherms. The parameter values in Table 1 indicate that POS, OS + P, and COS + P exhibit the strongest fluoride sorption, and all isotherms (except fluoride sorption to OS) are strongly nonlinear and concave down. The addition of phosphoric acid to OS and COS increased fluoride sorption substantially. Indeed, OS is the weakest sorbent for fluoride of all combinations studied by OS + P is the best sorbent.

**Table 1:** Parameters for Freundlich fluoride-sorption isotherms for all sorbents and conditions.

Parameters	*K*_*f*_ (L/g)	1/*n*	*R*^2^
Sorbents			
OS	0.0015	2.16	0.98
COS	0.79	0.73	0.99
POS	12.44	0.13	1.00
OS + P	17.70	0.27	1.00
COS + P	9.16	0.38	0.96
Hydroxyapatite^8^	10.50	0.99	0.99
Limestone^19^	5.85	0.59	0.96
Bone Char^20^	2.57	0.33	1.00
Acidic alumina^25^	4.17	0.31	0.92
CeO_2_–ZrO_2_ nanocages^26^	12.60	0.69	0.93
Hydroxyapatite-modified activated alumina^27^	2.79	0.60	0.98
Alumina nanoparticles^28^	1.54	0.59	0.98
Alumina oxide^29^	31.40	0.33	0.78
Kaolinite^30^	0.54	0.41	0.98
Modified alumina^31^	0.57	0.55	0.95
Boron nitride nanosheets^32^	1.96	0.41	0.96

The sorption data in Table 1 are consistent with other reported sorption data for fluoride. Gogoi and Dutta^19^ reported Freundlich *K*_*f*_ and 1/*n* parameters of 5.85 L/g and 0.59, respectively for fluoride sorption to limestone treated with 0.9 M phosphoric acid. They also reported that *K*_*f*_ increases and 1/*n* decreases when the concentration of phosphoric acid is increased. Similarly, Fan *et al*.^8^ reported *K*_*f*_ and 1/*n* values of 10.5 L/g and 0.99 for fluoride sorption to hydroxyapatite. These values changed to 12.4 L/g and 0.13 when the sorbent was changed to calcined oyster shells with added 4 M phosphoric acid.

### Mechanism of fluoride removal

**Figure 16:**
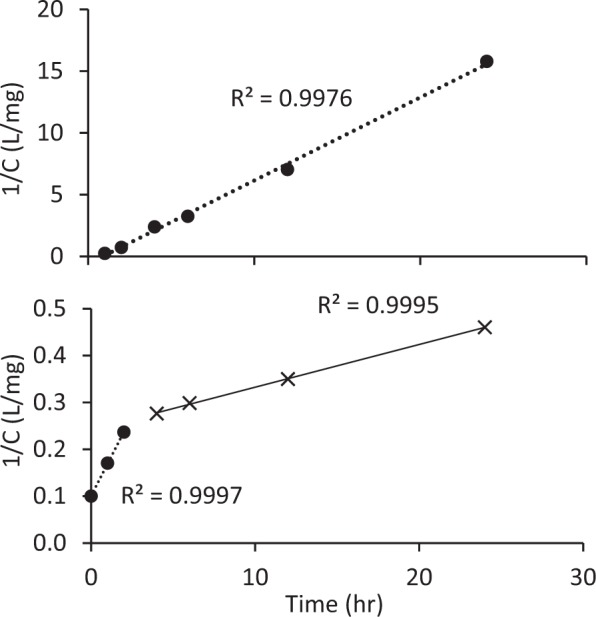
Second-order plot of fluoride sorption kinetics for OS + P at pH 7 and COS + P at pH 8 (Initial fluoride concentration was 10 mg/L).

To better understand that rate of fluoride sorption to OS + P and COS + P with an initial fluoride concentration of 10 mg/L, data were plotted according to a second-order rate process (Fig. 16). Tests of sorption kinetics were conducted at each 1 g/L of OS and COS. The samples were added with phosphoric acid to be 1.6 mM. These tests were conducted in triplicate and mean concentrations of fluoride were used. Sorption to both materials followed second-order reactions in agreement with other researchers^17,20^. The sorption rate constant *k* (L/mg-h) for OS + P was calculated as the slope of the graph in Fig. 16 (0.67). For COS + P, the plot was not linear over the whole time range; instead, a two-stage process was observed as the sorption rate decreased after 2 hr. The sorption rate constant *k* (L/mg-h) of COS + P was 0.068 for the first 2-hr period and 0.009 thereafter. Fluoride sorption to OS + P was more rapid than sorption to COS + P for all times. Yakup and Soboyeho^21^ and Chen *et al*.^22^ reported three-stage kinetic sorption of fluoride to granular ceramics. These results suggest that fluoride sorption is complex and may be influenced by multiple rate-limiting steps, including surface sorption and intraparticle diffusion.

The sorption mechanism for OS and COS is a chemical reaction between Ca^2+^ and F^−^ to make CaF_2_; ion exchange is the mechanism for POS which has hydroxyapatite that can make fluorapatite after reacting with F^−^. For the other sorbents (OS + P and COS + P), calcium (Ca^2+^) will be released and react with phosphoric acid to form hydroxyapatite and after exchanging F^−^ it is then converted to fluorapatite. The other mechanism involved could be calcium from the sorbent reacting with phosphate and fluoride to form fluorapatite. The sorption or exchange of fluoride by the calcium phosphates is the expected mechanism reported by other researchers. Gogoi *et al*.^15^ studied fluoride removal by Phosphoric acid-enhanced limestone defluoridation

To better know the reasons for the different sorption kinetics about fluoride sorption to OS + P and COS + P, a final set of experiments were conducted. For these experiments, the sorbent (either OS or COS) was combined with 10 mM phosphoric acid solution and equilibrated for 24 hours. Then the solid and liquid phases were separated by centrifugation and decanting of the supernatant. Fluoride was added to the supernatant in one batch reactor (herein referred to as the “liquid” reactor). A 10 mM phosphoric acid solution and fluoride were added to the solid phase fraction (herein referred to as the “solid” reactor. These two reactors were then equilibrated for 24 hr and centrifuged. The supernatant from both reactors was sampled and analyzed for F^−^.

**Figure 17:**
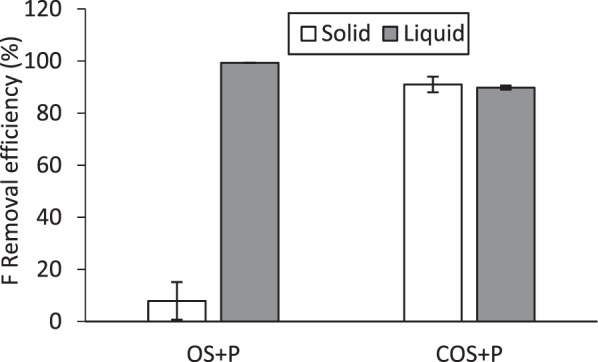
Fluoride removal in solid and liquid phases at 10 mM of phosphoric acid and fluoride concentration of 10 mg/L.

The percent reduction of fluoride added for the liquid and solid reactor for both OS and COS are shown in Fig. 17. For OS, there is negligible sorption of fluoride to the solid phase after it has essentially been washed by the 10 mM phosphoric acid solution. By contrast, the fluoride added to the wash solution is completely removed following equilibration and centrifugation of the “liquid” batch reactor. The reason for this is not completely certain at this time and likely will require additional experimentation. However, it is possible that during the washing of OS, calcium ions are released into the 10 mM phosphoric acid solution, and after subsequent addition of fluoride, Ca_5_(PO_4_)_3_F forms as a solid precipitate, which is then removed during centrifugation. The original solid-phase OS has little sorption capacity after the calcium ions have been removed by the phosphoric acid.

For COS, both the “liquid” and “solid” reactors demonstrate strong removal of fluoride (Fig. 17). This suggests that some of the calcium remains with the solid-phase sorbent and some is released into solution after washing with phosphoric acid solution. Therefore, formation of Ca_5_ (PO_4_)_3_F may be occurring both in solution (the liquid reactor) and on the solid surface (the solid reactor).

This observation may also support the different reaction kinetics observed by OS + P and COS + P. Fluoride reaction with sorbents in solution may be fast relative to reaction on the solid surface, so for OS + P, sorption is relatively rapid and the rate does not change with time because the precipitation reaction is occurring in the solution phase (Fig. 16). For COS + P, sorption occurs relatively quickly at early times because there is a solution-phase reaction. At later times, sorption is slower because it is caused primarily by a slower sorption process at the solid surface (Fig. 16). One prior study has reported that fluoride uptake on hydroxyapatite can occur like kinetics^2^ of COS + P. Conclusively, this novel sorbent has the potential for fluoride sorption from water and comprehensive experiments were conducted to explore this sorbent for fluoride sorption and to understand the underlying mechanism in the present study. There are some studies on fluoride removal with oyster shells and calcined oyster shells, but there is no research with oyster shells and phosphoric acid for fluoride removal. Material modification (with addition of phosphoric acid to form OS + P and COS + P) is the unique and novel idea for the given research. However, to date, no studies have investigated the equilibrium and kinetic sorption of fluoride with oyster shells treated to optimize performance.

Reusability is an important step to develop a cost-effective sorbent. Preliminary desorption experiments with fluoride loaded sorbent were conducted to explore desorption efficiency. Initial results show that the recyclability of the sorbent is possible and more comprehensive experiments at different pH values are underway. Some other researchers showed good reusability potential for fluoride removal. For example, Gogoi *et al*.^15^ explained that PAELD (Phosphoric acid-enhanced limestone defluoridation) technique, which uses easily available limestone and common soft beverage ingredient phosphoric acid, as an efficient, low-cost, safe, environment-friendly, simple, technique potential for rural application for fluoride removal from groundwater. The applicability of this novel sorbent in the field is also an important consideration. However, further investigation is warranted to find the field application of this technique and reusability potential of waste oyster shells. Future work will investigate this point.

## Materials and Methods

### Materials

Discarded oyster shells were collected from a local seafood restaurant and ground to ≤100 µm particle size with a Los Angeles abrasion machine to produce oyster shell powder (OS). A subset of the OS was heated at 700 °C for 3 hr to produce calcined oyster shell powder (COS). To adjust the calcium and phosphorus (Ca/P) ratio to 1.5, required phosphoric acid was added assuming that the COS is 100% CaO. The pH of this reaction was maintained around 10 by addition of NaOH. Once the reaction was complete, the sorbents were washed with deionized water, and dried for 48 hr at 105 °C to produce phosphoric-acid-treated oyster shell power (POS).

### Sorption studies

All batch sorption experiments were conducted in 50-mL glass test tubes. During equilibration, the reactors were mechanically shaken at 250 rpm at 20 °C. Fluoride concentration was measured with increasing dose of OS and COS sorbent. OS and COS dose varied from 1 g/L to 30 g/L whereas POS dose varied from 0.2 g/L to 2 g/L. Initial pH was adjusted to 7 ± 0.1. In order to find the optimum pH range, pH was changed from 4 to 10 with NaOH and H_2_SO_4_. 2 g/l of POS and 10 g/L of both OS and COS were used. Other conditions of both experiments were used 10 mg/L of fluoride concentration and 24 hr of contact time. Sorption isotherm tests were conducted with 100 mg/L of F^−^ and 48 hr of reaction time. Adsorbent amounts were changed from 1 g/L to 50 g/L. Sorption kinetic tests were conducted with 10 mg/L of fluoride and 1 g/L of adsorbent materials for POS. It was measured over 24 hr. The pH was adjusted to initial pH 7 ± 0.1 for POS.

### Characterization study

A PHI Versaprobe III X-ray photoelectron spectrometer (XPS; base pressure ~10^−8^ Pa) was utilized to identify and quantify fluorine present on the surface of processed POS; elemental concentrations were then compared with the unprocessed POS surface composition. Loose powder was mounted on the sample platen by first sprinkling and then gently pressing it with a SS spatula onto double-sided permanent Scotch tape; additional material was added until coverage was >99% by eye. XPS provides highly, surface-specific information, so coverage of only ~10 monolayers is required eliminate signal from the underlying adhesive. The before and after samples were separated by about 8 mm to minimize any surface diffusion effects. Data was taken by scanning a 100 micron X-ray spot over 1.4 mm, and averaged to eliminate artifacts that might appear with selection of information from a single spot.

XPS analysis provides accurate, surface-specific (~5 nm), elemental atomic composition of materials with a sensitivity of <0.1%, and is thus well-suited to determine the surface fluorapatite concentration in the processed POS. In XPS, incident monochromatic Al X-rays eject core-level electrons from the material, and the kinetic energy of the emitted photoelectrons is measured in a hemispherical electron energy analyzer. The binding energy of each photoelectron is then derived from these values as: BE = photon − KE − WF, where the WF is the work function of the electron spectrometer. Quantification is determined by comparison of the area under each photoelectron peak, normalized by a sensitivity factor. Survey spectra were acquired in fixed analyzer transmission mode (pass energy = 140 eV; step = 0.5 eV; 50 ms/step); an electron flood gun in conjunction with a low-energy ion gun maintained surface neutralization. The chemical nature of the fluorine bond was also investigated, running the Veraprobe at lower pass energy (pass energy = 26 eV) for improved energy resolution and with a smaller step (0.2 eV). High resolution spectra were collected for the F(1 s), C(1 s), Ca(2p), P(2p), and O(1 s) photoelectron features, as well as the F KVV Auger peak, for chemical analysis and comparison with standard published spectra for fluorapatite^23^.

Imaging and analysis of materials was performed with field emission scanning electron microscopy using a Thermo Fisher Scientific Helios UC G4 Dual Beam FIB-SEM equipped with an Oxford X-maxN energy-dispersive X-ray spectroscopy detector (EDX). For each sample, a small amount of powder was put on standard carbon tape ensuring that all particles stick to the tape. Small amounts of the powders were deposited on carbon tapes ensuring limited sample charging, so no conductive coating was used for EDX, and imaging was performed in the high vacuum mode after gold Palladium coating (2 nm thick). For imaging, a 5 kV electron beam energy with a 0.1 nA current was used in the field-free mode below 15000x and in the immersion mode above. For EDX analysis, a beam energy of 5 kV and electron beam energy with a 13 nA current was used.

The crystalline phases of the samples were examined by X-ray diffractometry (CuKa radiation, 40 kV/40 mA) on a Panalytical Empyrean diffractometer equipped with Bragg-Bretano HD (BBHD) incident beam optics and a Galipix3D detector operating in scanning line mode. The 2theta scan range was 10–70 degrees, and each measurement was the sum of three repeated scans. The quantification was done by the automated Rietveld refinement within Panalytical’s HighScore Plus software.

Cations (potassium, sodium, calcium, and magnesium) and anions (phosphate, sulphate, bromide, nitrite and chloride) were analyzed using a Dionex (Waltham, Massachusetts) ICS5000þ ion chromatograph connected to an auto sampler. The Dionex ICS5000þ system uses a patented eluent generation (EG) technology. Working solutions (5, 10, 20, and 50 mg/L) to calibrate the instrument were prepared from a multi-element stock solution for cations and anions. Stock solutions of the anions and cations were purchased from Thermo Fisher. Samples were prepared by filtering through a Whatman (Hampton, New Hampshire) syringe filter of 0.45 μm. Analytical blanks (Milli-Q water, Hampton, New Hampshire) and check standards were used at 10% for QA/QC purposes^24^.

### Addition of phosphoric acid

Phosphoric acid was added to OS and COS experimental batch reactors to produce OS + P (oyster shell with phosphoric acid) and COS + P (calcined oyster shell with phosphoric acid). All of these experiments were conducted with 50-mL batch reactors mixed at 250 rpm at 20 °C. In order to evaluate the effect of sorbent dose of OS and COS, the dosages were varied from 0.5 g/L to 10 g/L. Phosphoric acid was added to produce a concentration of 1.6 mM for OS + P solution and 3.2 mM for COS + P with 1 M of phosphoric acid. pH was adjusted to 7 ± 0.1 for OS + P and 8 ± 0.1 for COS + P.

### Sorption mechanism

In order to evaluate the effect of the molar ratio of phosphoric acid to F^−^, ratios from 1.5 to 12 were studied. Sorbent masses were 1 g/L for OS + P and 2 g/L for COS + P. pH was adjusted to 7 ± 0.1 for OS + P and 8 ± 0.1 for COS + P. In order to find optimum pH, pH was changed from 4 to 10 with NaOH or H_2_SO_4_. 1 g/L of OS and COS was used. Phosphoric-acid was adjusted to be 10 mM with 1 M of phosphoric acid. Reaction time of all of these tests was 24 hr. Sorption isotherm tests were conducted with a fluoride concentration of 100 mg/L and a reaction time of 48 hr. In order to form fluorapatite, theoretically 1 mole of fluoride is needed for 3 moles of phosphate. The phosphoric acid (PA) dose was adjusted to a PA/F molar ratio of 3. Sorbent dose was varied from 1 g/L to 15 g/L. Sorption kinetic tests were conducted with 10 mg/L of fluoride and 1 g/L of OS and 2 g/L of COS. pH was adjusted to 7 ± 0.1 for OS + P and 8 ± 0.1 for COS + P. Fluoride concentration was quantified as a function of time up to 24 hr. These tests were performed in triplicate for OS + P and COS + P.

To further explore the mechanism of fluoride removal, the following experiments were performed using OS and COS. The concentration was adjusted to 1 g/l as 0.05 g of OS and COS put in 50 ml of tubes. The sorbent (either OS or COS) were combined with a 10 mM phosphoric acid solution and equilibrated for 24 hr. The samples were centrifuged and the supernatant was decanted from the solid phase. The separated solid phase was combined with 10 mM phosphoric acid solution and fluoride. Fluoride was also added to the decanted liquid phase. Both systems were then equilibrated for an additional 24 hr. Both solutions were centrifuged, and the supernatant was analyzed for F^−^ concentration.

### Fluoride ion measurement

Fluoride concentration in water was quantified using a calibrated fluoride specific-ion electrode (ORION 9609BNWP) with a pH/mV meter (sensIONTM, MM340). Standard solutions used for calibration were made from NaF. Fluoride was analyzed with a total ionic strength adjustment buffer (TISAB II).
